# Chemical engineering of quasicrystal approximants in lanthanide-based coordination solids

**DOI:** 10.1038/s41467-020-18328-5

**Published:** 2020-09-17

**Authors:** Laura Voigt, Mariusz Kubus, Kasper S. Pedersen

**Affiliations:** Department of Chemistry, Technical University of Denmark, Kemitorvet, Building 207, DK-2800 Kgs Lyngby, Denmark

**Keywords:** Coordination chemistry, Metal-organic frameworks, Structure of solids and liquids

## Abstract

Tessellation of self-assembling molecular building blocks is a promising strategy to design metal-organic materials exhibiting geometrical frustration and ensuing frustrated physical properties. Appearing in two-dimensional quasiperiodic phases, tilings consisting of five-vertex nodes are regarded as approximants for quasicrystals. Unfortunately, these structural motifs are exceedingly rare due to the complications of acquiring five-fold coordination confined to the plane. Lanthanide ions display the sufficient coordinative plasticity, and large ionic radii, to allow their incorporation into irregular molecule-based arrays. We herein present the use of ytterbium(II) as a five-vertex node in a two-dimensional coordination solid, YbI_2_(4,4′-bipyridine)_2.5_. The semi-regular Archimedean tessellation structure verges on quasicrystallinity and paves the way for lanthanide-based metal-organic materials with interesting photonic and magnetic properties.

## Abstract (web summary)

Tessellation of self-assembling molecular building blocks is attractive for accessing metal-organic materials with geometric frustration, however such motifs are rare. Here the authors use ytterbium(II) as a five-vertex node to assemble an Archimedean tessellation in a bulk, molecule-based material.

## Introduction

**Fig. 1:**
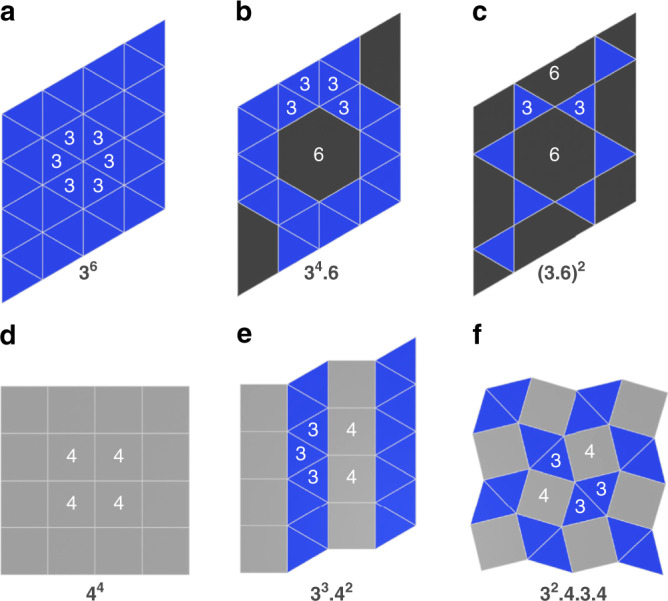
Archimedean tessellations. Six of the 11 regular (**a**, **d**) and semi-regular (**b**, **c**, **e**, **f**) Archimedean tessellations of the Euclidean plane: **a** triangular, **b** snub hexagonal, **c** trihexagonal (kagomé), **d** square, **e** elongated triangular, and **f** snub square tiling.

Quasicrystals are ordered materials lacking translational symmetry^1^, which leads to unique physical properties^2^, including novel magnetic and photonic phenomena^3–5^. They exhibit symmetries that are forbidden for any periodic structure, such as the 5-fold rotational symmetry first discovered in an Al–Mn alloy by Shechtman et al.^6^ or the 12-fold symmetry in dodecagonal quasicrystals^7^. Dodecagonal symmetry is achieved in a maximally random assembly of squares and triangles linked through four-, five-, and six-vertex nodes^8,9^. Dodecagonal quasicrystallinity has been observed in a plethora of materials including alloys^10,11^, mesoporous silica^12^, nanoparticle superlattices^13^, and copolymers^14^. Notably, all of these quasicrystalline phases have motifs of, or coexist with, domains of periodic tilings of squares and triangles, whose existence was hypothesized by Frank and Kasper^15^, and whose topologies were first introduced by Kepler in *Harmonices Mundi* in 1619^16^. The tilings of squares and triangles belong to the uniform tessellations of the two-dimensional (2D) (Euclidian) plane^17^, where all vertices are identical and the tilings are labeled by the sequence of their surrounding polygons. For instance, six-vertex nodes are found only in the regular triangular tiling (3^6^; Fig. 1a) containing a single equilateral polygon, whereas five-vertex nodes are part of the snub hexagonal (3^4^.6), elongated triangular (3^3^.4^2^), and snub square tilings (3^2^.4.3.4; cf. Fig. 1b, e, f). Due to their similarities with the local structure of dodecagonal quasicrystals, crystalline phases with structure motifs of five-vertex semi-regular Archimedean tessellations (ATs) are referred to as quasicrystal approximants^9,12,18–20^. The appearance of approximants suggests the possibility of designing quasicrystallinity by chemical means. The realization of regular tilings in chemistry and nature is ubiquitous. However, of the ATs only the trihexagonal tiling ((3.6)^2^; cf. Fig. 1c), the kagomé lattice, appears frequently^21^. Notably, ATs in materials have been hailed as a key component for exploring novel photonics applications^22–24^ and to host peculiar magnetic phenomena, such as frustration^25–28^, which call for novel synthetic strategies to their authentication. Whereas for intermetallics^29^ numerous quasicrystalline approximants have been isolated, ATs have remained almost elusive in metal-organic materials. Reticular coordination chemistry harbors the synthetic handles on controlling dimensionality and topology of metal-organic coordination solids, suitable for targeting complex tessellations. Indeed, applying this strategy resulted in a singular example of a metal-organic quasicrystal consisting of a surface self-assembled array of Eu atom nodes linked by ditopic carbonitrile bridging ligands^7^. This network is reminiscent of a random tiling containing nanoscale motifs of the semi-regular ATs. The flexibility and the large atomic radii of Eu allowed for coordinating up to six ligands in the equatorial plane allowing the formation of local dodecagonal symmetry^7^. Notably, small differences in the preparation conditions led to the formation of prevailing 3^3^.4^2^ or 3^2^.4.3.4 tilings, which underlines the close relationship of the periodic approximants with the dodecagonal phase. Accordingly, the assembly of atomic Ce and the same ditopic ligand on a Ag(111) surface lead to the formation of a 3^2^.4.3.4 tiling^30^. Only recently, an ionic liquid-based approach led to the isolation of a bulk coordination solid containing layers of [(UO_2_)(1,2,4-triazolate)_2.5_]^(1/2)−^, constituting the first example of an 3^2^.4.3.4 tiling realized through fivefold coordinated *trans*-{UO_2_}^2+^ nodes^20^. Herein, however, the presence of a net charge of the 2D layer could impede the possibilities for further processing of this material for nanostructuring and the nonmagnetic uranyl nodes hinder the exploration of magnetic properties. Similar to uranium, the lanthanide ion series distinguishes itself by a strong propensity to high coordination numbers. In addition, the lanthanide ions exhibit paramount plasticity in their chemical bonding and unique optical and magnetic properties, of immense technological importance, which are not paralleled by any other elements^31^. Motivated by the possibilities to extend these properties to ATs and quasiperiodic materials, we, herein, report a coordination-assembly strategy and the crystallographic characterization of the first example of a lanthanide-based AT in a bulk, molecule-based material.

## Results

### Structural characterization and description

**Fig. 2:**
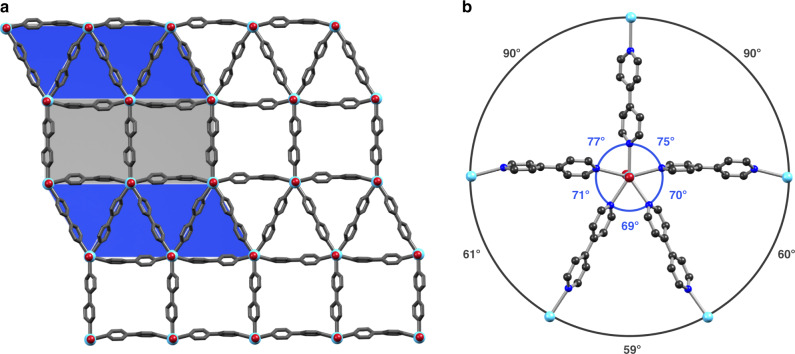
Structure of 1. Representations of the crystal structure of **1** as determined by single-crystal X-ray diffraction at *T* = 120(1) K. **a** Fragment of the constituent coordination layers with an overlay of the ideal 3^3^.4^2^ tessellation. **b** The five-vertex node of *trans*-{YbI_2_} and bipy linkers in **1**. The ∠N–Yb–N and ∠Yb–Yb–Yb are shown in blue and gray, respectively. Selected bond lengths (Å, e.s.d.): Yb–I 3.1210(4), 3.1321(4), Yb–N 2.564(4)–2.633(4). Yb···Yb separations (Å) amount to 12.14 and 12.25 for the squares, and 12.32, 12.14, and 12.34 for the triangles (Yb light blue; I dark red; N blue; C gray; hydrogen atoms and co-crystallized CH_3_CN have been omitted for clarity).

**Fig. 3:**
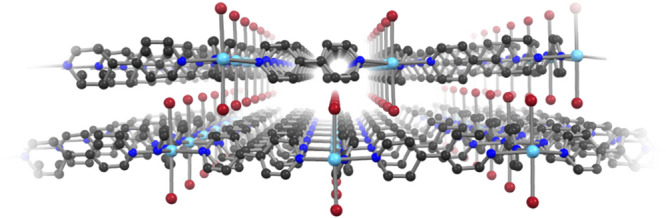
Perspective view of the 2D nature of 1. Layers of **1** in which the iodide ligands point towards the cavities of the adjacent layers. ∠I–Yb–I 175.60(1)°. (Yb, light blue; I, dark red; N, blue; C, gray; hydrogen atoms and co-crystallized CH_3_CN have been omitted for clarity).

Numerous examples of first-row transition element-based 2D coordination solids pillared on 4,4′-bipyridine (bipy) and related ditopic, organic ligands are known^32^. Therein, the limiting, prevailing coordination number of six imposes a square (4^4^; cf. Fig. 1d) tessellation of the 2D plane calling for employment of significantly larger metal ions for the generation of ATs. The tendency to high coordination numbers in di- and trivalent lanthanide (Ln(II/III)) compounds offers the possibility of generating, for instance, [LnI_2_(pyridine)_5_]^+^^/^^0^ (Ln = Lu^33^, Sm^34^). Notably, these systems closely resemble a local *D*_5h_ coordination geometry of the Ln(II/III) with the organic ligands placed in the equatorial plane, presumably directed by the space-filling, axial iodide ligands. Translating this structure-directing motif to 2D coordination solids seems key for tailoring metal-organic ATs and quasicrystals. Indeed, the self-assembly reaction of ytterbium(II) iodide with bipy in acetonitrile at room temperature under strictly anaerobic conditions yielded dark blue, block-shaped crystals of YbI_2_(bipy)_2.5_·1.5 CH_3_CN (**1**) suitable for single-crystal X-ray diffraction analysis. Compound **1** crystallizes in the triclinic space group *P*$\bar 1$ hosting a single Yb in the asymmetric unit (Fig. 2 and Supplementary Fig. 1). The {YbN_5_} moiety deviates marginally from planarity with a maximal distortion from ideality of ∼5% for I2–Yb–N3 and approximates closely a *D*_5h_ coordination environment with N–Yb–N angles in the range from 69° to 77°, close to the ideal angle of 72° (Fig. 2b). Within the 2D plane, the linking of bipy and Yb(II) forms both square and triangular tiles with the bipy linkers spanning the edges. The squares and triangles closely approximate equilateral geometries with edge lengths of 12.14 Å and 12.25 Å for the squares and 12.32 Å, 12.14 Å, and 12.34 Å for the triangles. Despite the local *D*_5h_ symmetry, a slight bending of the bipy linkers leads to the formation of an ideal 3^3^.4^2^ tessellation (Fig. 2a). The 2D layers are stacked with an inter-plane separation of ∼6 Å imposed by the axial iodide ligands (Fig. 3).

The packing motif of the 2D layers leads to the formation of solvent-accessible pore channels of distorted 3^3^.4^2^ topology along both the crystallographic *a* and *b* directions and of distorted 3^6^ topology along the *c* direction (Supplementary Fig. 2). To our knowledge, **1** is the first example of any coordination solid constructed from divalent lanthanide ions. The presence of potentially reducible bipy and strongly reducing Yb(II) (*E*^⦵^_Yb_^3+^_/Yb_^2+^ = –1.1 V) could be expected to lead to the occurrence of redox events, similar to what is found in the reaction between elemental sodium and bipy^35^. However, the single-crystal structure revealed Yb–N bond lengths between 2.564(4) Å and 2.633(4) Å and Yb–I bond lengths of 3.1210(4) Å and 3.1321(4) Å, which are close to those found in the related [Yb^II^I_2_(3,5-lutidine)_4_] (Yb–N 2.586(3) Å, Yb–I 3.132(1) Å)^36^, and [Yb^II^I_2_(CH_3_CN)_5_] (Yb–N 2.537(2)–2.566(3) Å, Yb–I3.1124(2), 3.1326(2) Å)^37^, and much further away from [Yb^III^I_2_(pyridine)_5_]I·1/2 pyridine (Yb–N 2.454(4)–2.482(3) Å, Yb–I 2.9645(3) 2.9729(3) Å)^38^.

**Fig. 4:**
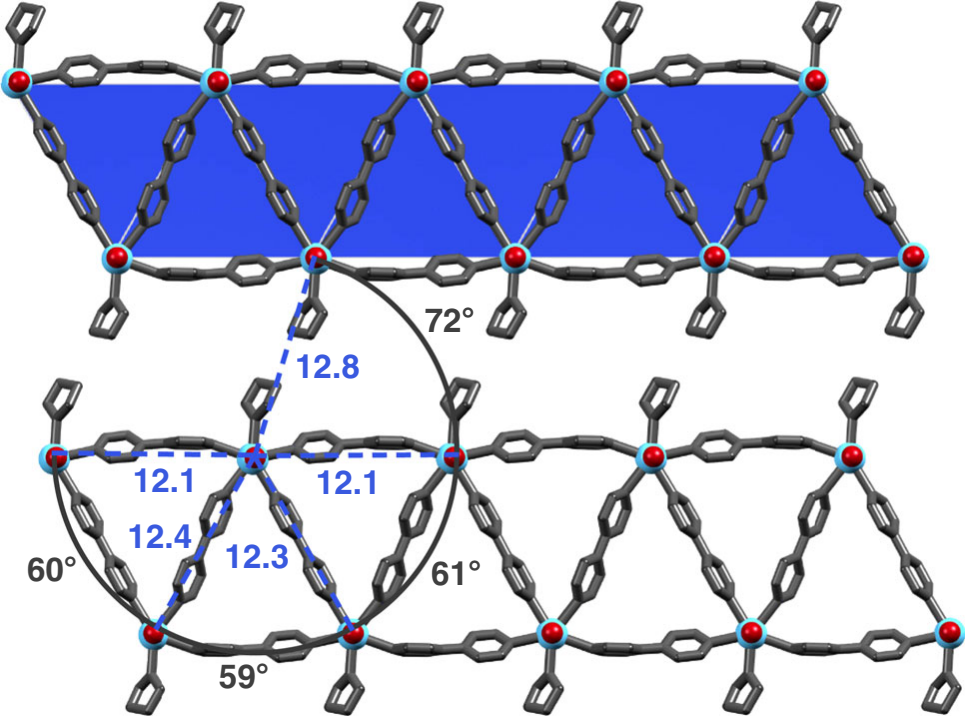
Structure of 2. Fragment of the structure of **2** as determined by single-crystal X-ray diffraction with indicated Yb···Yb separation lengths (Å) and ∠Yb···Yb···Yb angles. Selected additional bond lengths (Å, e.s.d.) and angles (°, e.s.d): Yb–I 3.1311(8), 3.1038(8); Yb–N 2.585(5)–2.666(5); Yb–O 2.454(5); ∠N–Yb–N 69.0(2)–71.9(2); ∠N–Yb–O 73.7(2)–74.1(2). (Yb, light blue; I, dark red).

The structural data thus echo the presence of Yb(II) and the absence of any Yb(III)–bipy^•^^–^ valence tautomer formation, which has been observed in related, molecular Yb(II) complexes of 2,2′-bipyridine^39,40^. Changing the solvent of the synthesis to tetrahydrofuran (THF) resulted in a slightly different compound, YbI_2_(bipy)_2_(THF)·1.5 THF (**2**). The dark green **2** is structurally related to **1** but lacks the square tiles due to coordination of a THF solvent molecule and can be considered to be composed of isolated strands of a bisected 3^6^ tessellation (Figs. 1a and 4, and Supplementary Fig. 3). Notably, the crystal packing enforces a supramolecular distorted 3^3^.4^2^ tessellation with interchain Yb···Yb···Yb angles of 72°, and similar inter- and intrachain Yb···Yb separations (12.8 Å vs. ~12.2 Å; Fig. 4). Interestingly, considering only the Yb sites, the structure can be described as resting at an intermediate position between the 3^3^.4^2^ (with quadratic tiles: ∠Yb···Yb···Yb = 90°) and the 3^6^ (∠Yb···Yb···Yb = 60°) tessellations. The Yb–I (3.1311(8), 3.1038(8) Å) and Yb–N (2.585(5)–2.666(5) Å) bond lengths are all close to those of **1**, indicating the presence of divalent Yb in **2**. The oxidation state assignment of Yb(II) and the absence of any valence-tautomerism in **1** and **2** were further corroborated by bulk magnetometry. Yb(II), having a closed-shell [Xe]4f^14^ electronic configuration, is expected to be fully diamagnetic. Indeed, the magnetic susceptibility-temperature products, *χT*, of both **1** and **2** at 273 K were typically vanishing at ∼0.06 cm^3^ K mol^–1^ (Supplementary Fig. 4). Assuming the identity of a paramagnetic impurity as Yb(III) (2.57 cm^3^ K mol^–1^ calculated for ^2^F_7/2_ and *g*_*J*_ = 8/7), its concentration is estimated to be <2%.

## Discussion

A quasicrystalline tiling consisting solely of squares and triangles has an optimal square-to-triangle ratio of √3/4 ≈ 0.43^8,41^. Hence, to approach a dodecagonal quasicrystalline tiling with the 3^3^.4^2^ tessellation structure at hand, triangle-rich motifs need to be introduced into the tiling. The lanthanide ions here stand out as ideal materials modules due to their exceptional structural versatility that is inimitable amongst the inner and outer transition metal elements of the periodic table. This allows their accommodation into the in-plane tetra-, penta-, and hexa-coordinated nodes that coexist in dodecagonal quasicrystals^7,12^. It is here particularly important that the strategy is not reserved for divalent lanthanide ions. Indeed, several molecular complexes of the trivalent lanthanide ions possess an approximate *D*_5h_ (e.g., [LnI_2_(THF)_5_]^+^ ^42–47^ and [LuI_2_(pyridine)_5_]^+^ ^33,38^) coordination environment necessary for their incorporation into such materials. Notably, contrasting the intermetallics, the typical optical transparency of lanthanide-based coordination solids provides for exploitation of optical properties of complex tilings. For instance, ATs offer the potential of photonic band gap opening^23,48^, which in conjunction with the unique luminescence characteristics of the lanthanide ions^31^, lays the foundation for materials with novel optical properties. In addition, the field of molecular magnetism has recently seen a blossoming of approximate *C*_5_ symmetric single-ion magnets with exceedingly high magnetization reversal barriers ^49–51^. Linking of single-ion magnets or lanthanide-based qubits^52^ into ATs and quasicrystals to yield spin-frustrated arrays would be an attractive extension of this work^53^. This approach also comprises controlling metal–ligand redox events with the aim of introducing electronic conductivity and increasing the strength of inter-ion magnetic interactions in ATs and derived network structures as has been developed for regular tessellations with lighter first-row transition element analogs^54^.

In conclusion, we have reported a chemical strategy to tailor AT topologies in coordination solids by employing the propensity for high coordination numbers intrinsic to the lanthanide series ions. The appearance of five-vertex tilings suggests the possibility for the occurrence of metal-organic quasicrystalline phases in a bulk material. Exemplified by the structure of **2**, our approach opens up a novel route to design supramolecular metal-organic AT analogs, which is currently pursued for fully organic materials^55–57^. In addition, the 2D nature hosts potential for nanostructuring and exfoliation of complex tilings, as is now intensively practiced for the 3d congeners with regular tessellation structures^58^. Although nanoscale arrays of lanthanide-based ATs have previously been observed on surfaces using scanning tunneling microscopy^30^, the isolation of **1** demonstrates the possibilities to design lanthanide-based ATs in bulk, crystalline materials. This result paves the way for next-generation materials with complex and non-periodic tiling structures hosting novel photonic and magnetic phenomena originating from the unique physical properties of the lanthanide ions.

## Methods

### Synthesis

All procedures were carried out in an InertLab glovebox with a dry Ar atmosphere. Dry and air-free solvents were obtained from a Puresolv MD 7 solvent purification system. YbI_2_ and 4,4′-bipyridine (bipy) were supplied by Sigma-Aldrich and were used as received. Elemental analyses were performed by the Mikroanalytisches Laboratorium Kolbe (Oberhausen, Germany). Synthesis of YbI_2_(bipy)_2.5_·1.5 CH_3_CN (**1**): YbI_2_ powder (160 mg, 375 µmol) was placed in a standard test tube (160 × 16 mm, soda glass). The solid was carefully covered with acetonitrile (18 mL) with a pipette, as to not swirl up the YbI_2_. An acetonitrile solution (3 mL) of bipy (293 mg, 1.88 mmol) was slowly layered on top of that and the reaction mixture was left undisturbed for 7 days. Dark blue crystals formed at the bottom of the reaction tube were isolated using a pipette and carefully washed with cold acetonitrile (−20 °C; 2 × 5 mL). Yield: 210 mg (65% based on Yb). Calculated elemental analysis (found) for C_25_H_20_YbI_2_N_5_, C_3_H_4.5_N_1.5_: C, 38.26% (36.67%); H, 2.81% (2.63%); I 28.88% (28.79%); N, 10.36% (9.51%). Synthesis of YbI_2_(bipy)_2_(THF)·1.5 THF (**2**): YbI_2_ (108 mg, 253 µmol) and bipy (216 mg, 1.38 mmol) were separately dissolved in THF (3.7 mL and 1.3 mL, respectively) and the YbI_2_ solution was added dropwise to the bipy solution. Afterwards the mixture was kept at −20 °C for 2 days. The dark green crystals formed at the bottom of the vial were filtered and washed with toluene (10 mL) and dried under vacuum. Yield: 177 mg (70% based on Yb). Calculated elemental analysis (found) for YbI_2_C_24_H_24_N_4_O_1_, C_6_H_12_O_1.5_: C, 39.19% (37.95%); H, 3.95% (3.66%); I 27.60% (28.59%); N, 6.09% (6.35%).

### Crystallography

Single crystals of **1** and **2** were covered with polybutene oil (Aldrich, >90%) and mounted onto a nylon loop, which was attached to a SuperNova Dual Source CCD diffractometer. Data were collected using Mo Kα radiation at *T* = 120(1) K. Using Olex2^59^, the structure was solved with the ShelXT^60^ structure solution program and refined with the SHELXS^61^ refinement package using least squares minimization. All non-hydrogen atoms were refined anisotropically (cf. Supplementary Table 1). The crystal structure of **1** consists of one additional, highly disordered solvent molecule in the unit cell, i.e., half a solvent molecule per formula unit, which could not be modeled accurately and was treated by solvent mask method implemented in Olex2^62^. Twenty-three electrons were found in a volume of 282 Å^3^ in one void per unit cell. This indicates the presence of one acetonitrile molecule per unit cell, which accounts for 22 electrons. Similarly, a solvent mask was calculated for **2** and 38 electrons were found in a volume of 177 Å^3^ in one void per unit cell. This is consistent with the presence of one molecule of THF per unit cell, which accounts for 40 electrons. Powder X-ray diffraction patterns (Supplementary Fig. 5) were measured at room temperature in transmission mode with a Huber G670 powder diffractometer using Cu Kα_1_ (*λ* = 1.5406 Å, quartz monochromator) radiation. The powder samples were measured in sealed bags and the powder of **1** was additionally immersed in polybutene oil (Aldrich, >90%) to suppress loss of co-crystallized CH_3_CN molecules. The resulting powder patterns were background corrected.

### Magnetization measurements

The isofield dc magnetization measurements were performed using the VSM option on a QuantumDesign Dynacool Physical Property Measurement System equipped with a 9T dc magnet in the temperature range from 1.7 K to 273 K in a magnetic field of 0.1 T. The polycrystalline samples were loaded into standard QuantumDesign powder capsules inside an Ar-filled glovebox. The experimental data were corrected for diamagnetic contributions from the sample holder and the intrinsic sample diamagnetism.

## Supplementary information

Supplementary Information

Supplementary Data 1

Supplementary Data 2

Supplementary Data 3

Supplementary Data 4

## Data Availability

The X-ray crystallographic coordinates for structures reported in this Article have been deposited at the Cambridge Crystallographic Data Centre (CCDC), under the deposition numbers CCDC 1988173–1988174. These data can be obtained free of charge from The Cambridge Crystallographic Data Centre via www.ccdc.cam.ac.uk/data_request/cif. All other relevant data are available from the authors on request.
