# Supplementary material for: Chemical engineering of quasicrystal approximants in lanthanide-based coordination solids
Source: Nat Commun. 2020 Sep 17;11:4705. doi: 10.1038/s41467-020-18328-5 (PMC7498582; doi:10.1038/s41467-020-18328-5)
Supplement: Supplementary file 1 — Supplementary Information [file 41467_2020_18328_MOESM1_ESM.pdf]

Chemical engineering of quasicrystal approximants in lanthanide-based coordination solids

Voigt *et al.*

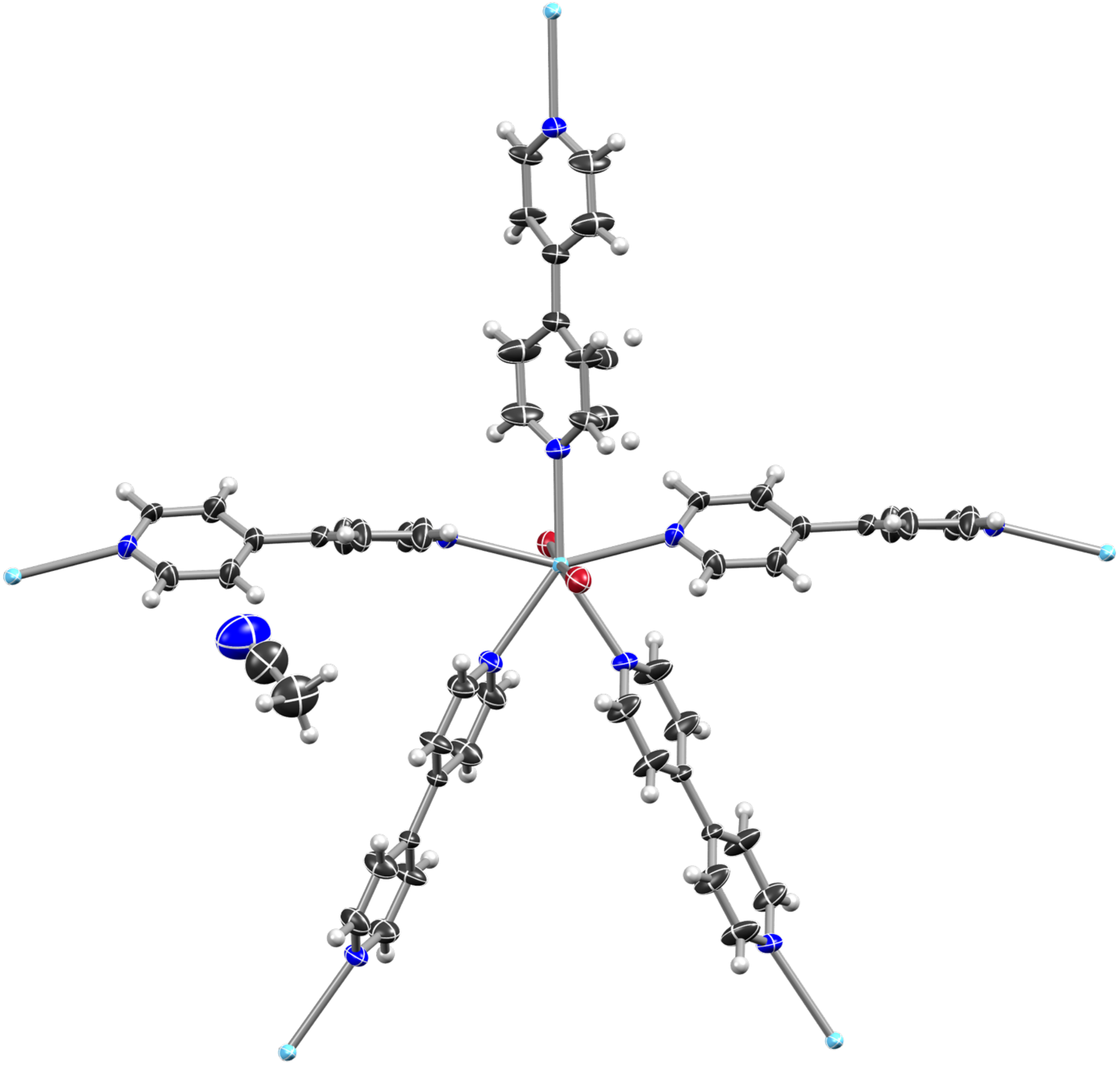

Supplementary Fig. 1. Thermal ellipsoid plot of **1** drawn at 60% probability level (Yb, light blue; I, dark red; N, blue; C, grey; H, white). One of the bipy ligand is slightly disordered over two positions.

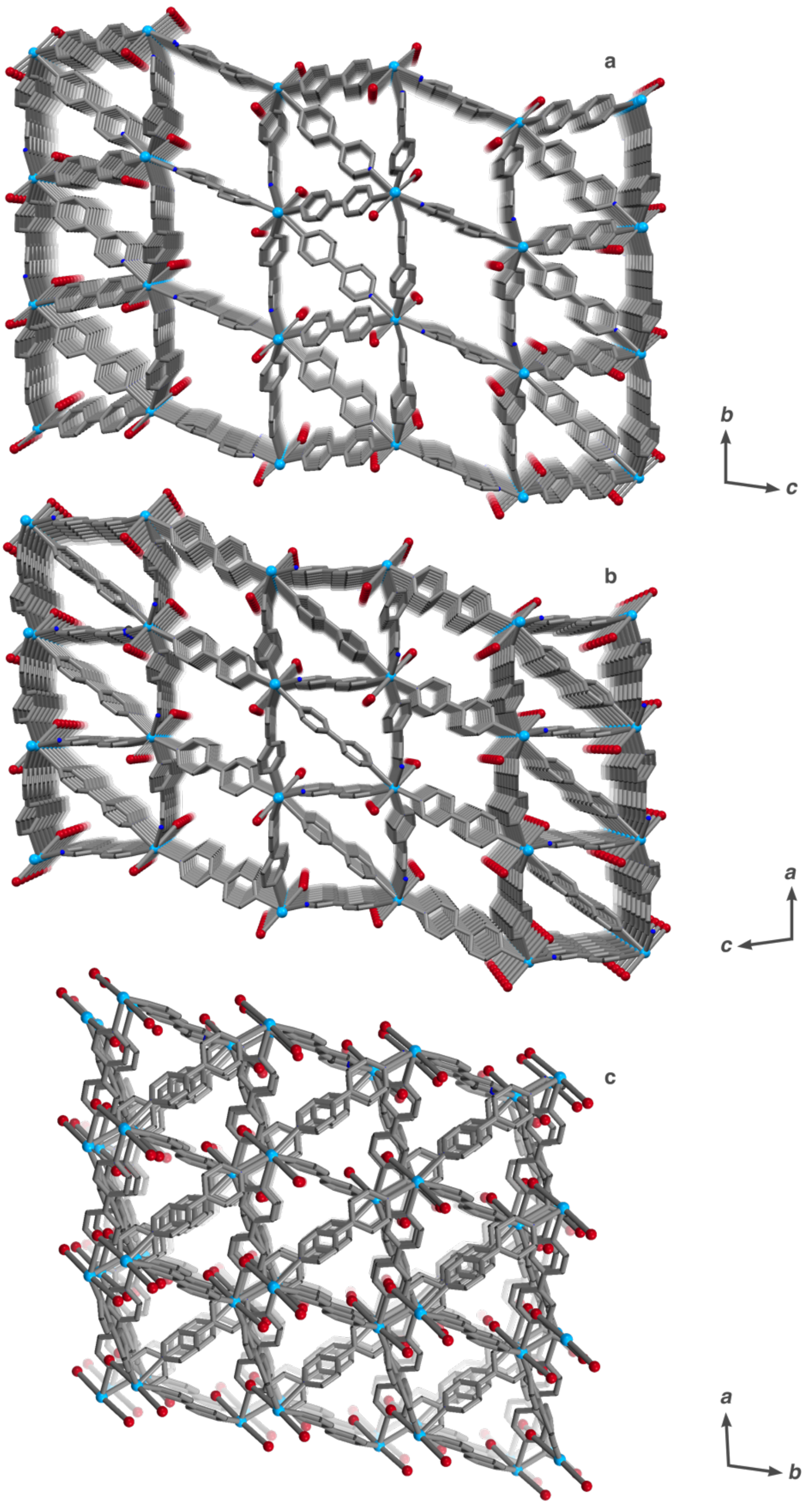

Supplementary Fig. 2. Packing motif of **1** shown along its three crystallographic directions (*a* top, *b* middle, *c* bottom), as determined from single-crystal X-ray diffraction. Solvent molecules and hydrogen atoms were omitted for clarity (Yb, light blue; I, dark red; N, blue; C, grey).

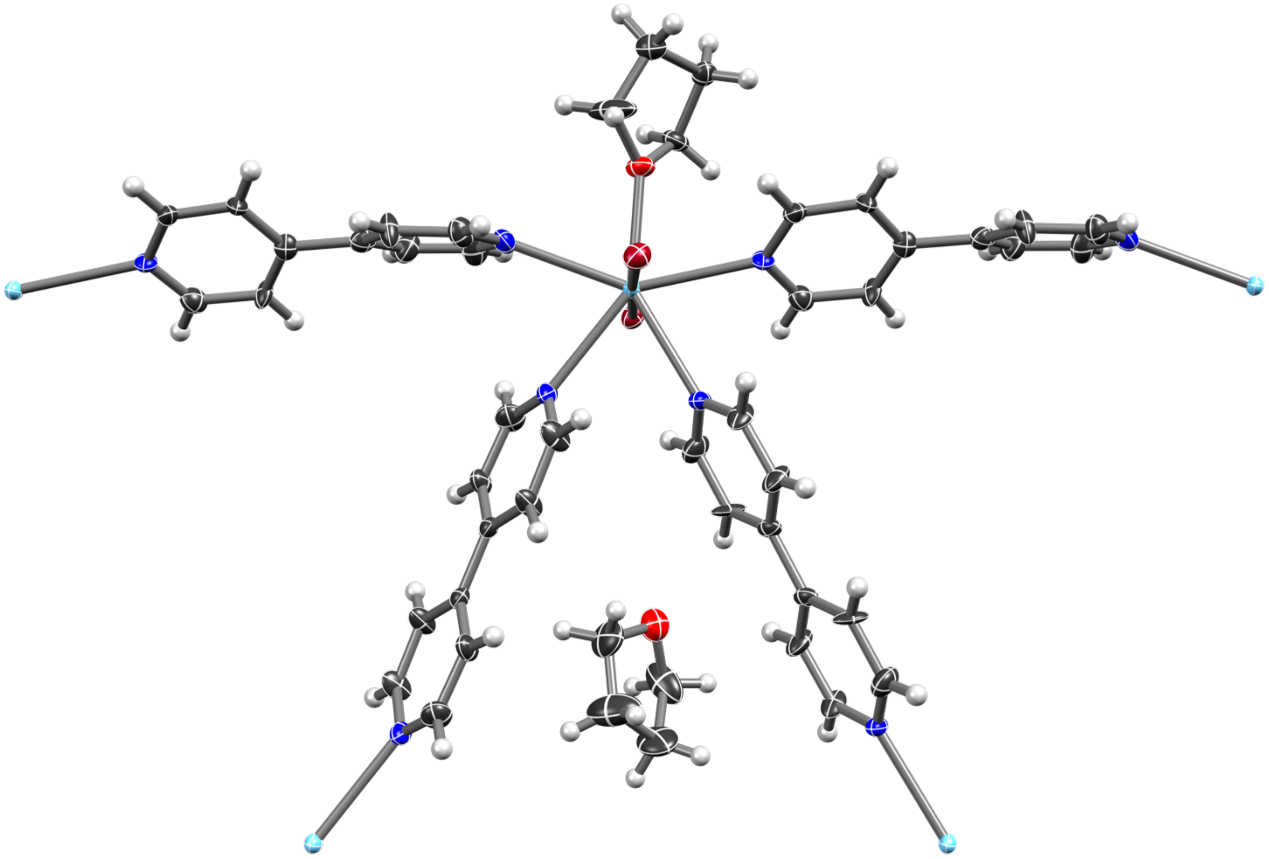

Supplementary Fig. 3. Thermal ellipsoid plot of **2** drawn at 60% probability level (Yb, light blue; I, dark red; O, red; N, blue; C, grey; H, white).

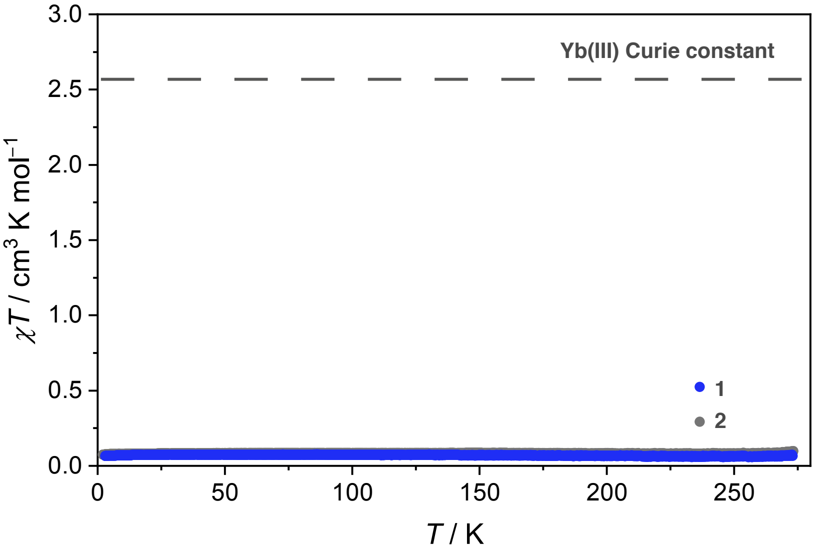

Supplementary Fig. 4. χT products ($\mu_0 H$ = 1000 Oe) of **1** and **2** and the theoretical χT value of Yb(III) shown for comparison.

Supplementary Table 1. Crystallographic information and refinement parameters of **1** and **2**.

Compound	**1**	**2**
CCDC number	1988174	1988173
Empirical formula	$C_{25}H_{20}YbI_2N_5$, C_2H_3N	$C_{24}H_{24}YbI_2N_4O$, C_4H_8O
Formula weight / g mol^{-1}	858.35	883.41
Temperature / K	120	120
Crystal system	Triclinic	Triclinic
Space group	$P\bar{1}$	$P\bar{1}$
a / Å	8.8824(3)	9.1540(6)
b / Å	9.9235(3)	12.1469(6)
c / Å	19.5290(5)	14.4851(8)
α / °	96.183(2)	94.419(4)
β / °	101.009(2)	91.510(5)
γ / °	99.783(2)	93.165(4)
Volume / Å^3	1647.55(9)	1602.64(16)
Z	2	2
ρ_{calc} / g cm^{-3}	1.730	1.831
μ / mm^{-1}	4.735	4.873
F(000)	806.0	840.0
Radiation	Mo Kα (λ = 0.71073 Å)	Mo Kα (λ = 0.71073 Å)
θ range for data collection / °	5.628–59.428	5.35–59.408
Index ranges	$-12 \leq h \leq 12$	$-11 \leq h \leq 11$
	$-12 \leq k \leq 13$	$-16 \leq k \leq 16$
	$-27 \leq l \leq 26$	$-19 \leq l \leq 13$
Reflections collected	15780	14460
Independent reflections	7798 [R_{int} = 0.0382]	7608 [R_{int} = 0.0536]
Data/restraints/parameters	7798/0/344	7608/0/334
Goodness-of-fit on F^2	1.043	0.946
Final R_1 index [$F^2 \geq 2\sigma(F^2)$]	0.0385	0.0490
Final wR_2 index [F^2]	0.0832	0.0629
Largest diff. peak/hole / e Å^{-3}	1.54/–1.21	1.32/–1.22

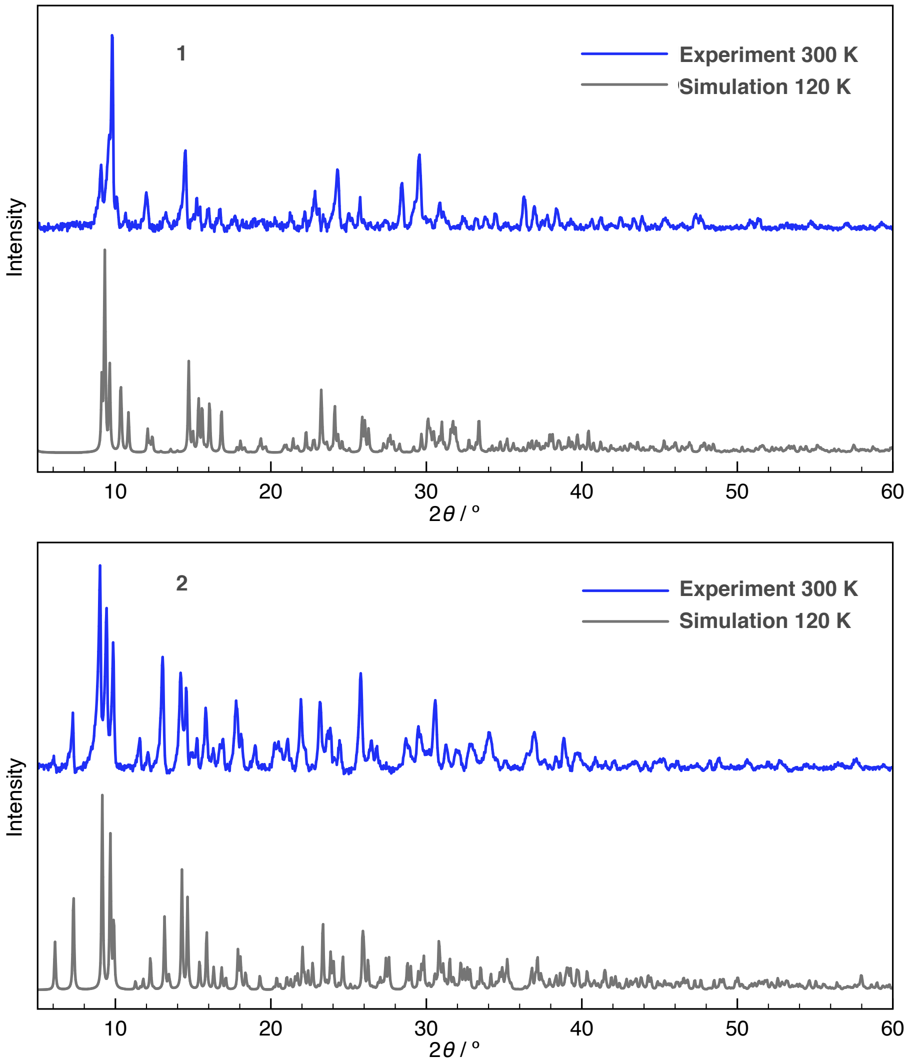

Supplementary Fig. 5. Room-temperature powder X-ray diffractograms of **1** (top) and **2** (bottom) and the simulated powder diffractograms obtained using the single-crystal X-ray structures measured at T = 120 K.
