# Supplementary material for: Chemical engineering of quasicrystal approximants in lanthanide-based coordination solids
Source: Nat Commun. 2020 Sep 17;11:4705. doi: 10.1038/s41467-020-18328-5 (PMC7498582; doi:10.1038/s41467-020-18328-5)
Supplement: Supplementary file 4 — Supplementary Data 3 [file 41467_2020_18328_MOESM4_ESM.pdf]

checkCIF (basic structural check) running

Checking for embedded fcf data in CIF ...
Found embedded fcf data in CIF. Extracting fcf data from uploaded CIF, please wait **. .**

checkCIF/PLATON (basic structural check)

Structure factors have been supplied for datablock(s) lv_ybi2_23_19

THIS REPORT IS FOR GUIDANCE ONLY. IF USED AS PART OF A REVIEW PROCEDURE FOR PUBLICATION, IT SHOULD NOT REPLACE THE EXPERTISE OF AN EXPERIENCED CRYSTALLOGRAPHIC REFEREE.

No syntax errors found. CIF dictionary
Please wait while processing .... Interpreting this report

Structure factor report

Datablock: lv_ybi2_23_19

Bond precision: C-C = 0.0079 A Wavelength=0.71073

Cell: a=8.8824(3) b=9.9235(3) c=19.5290(5)
alpha=96.183(2) beta=101.009(2) gamma=99.783(2)

Temperature: 120 K

	Calculated	Reported
Volume	1647.56(9)	1647.55(9)
Space group	P -1	P -1
Hall group	-P 1	-P 1
Moiety formula	C25 H20 I2 N5 Yb, C2 H3 N [+ solvent]	C25 H20 I2 N5 Yb, C2 H3 N
Sum formula	C27 H23 I2 N6 Yb [+ solvent]	C27 H23 I2 N6 Yb
Mr	858.35	858.35
Dx,g cm-3	1.730	1.730
Z	2	2
Mu (mm-1)	4.735	4.735
F000	806.0	806.0
F000'	803.62	
h,k,lmax	12,13,27	12,13,27
Nref	9362	7798
Tmin,Tmax	0.629,0.910	0.785,1.000
Tmin'	0.617	

Correction method= # Reported T Limits: Tmin=0.785 Tmax=1.000
AbsCorr = MULTI-SCAN

Data completeness= 0.833 Theta(max)= 29.714

R(reflections)= 0.0385(6207) wR2(reflections)= 0.0832(7798)

S = 1.043 Npar= 344

The following ALERTS were generated. Each ALERT has the format
test-name_ALERT_alert-type_alert-level.
Click on the hyperlinks for more details of the test.

Alert level C

PLAT213_ALERT_2_C Atom C19 has ADP max/min Ratio ..... 3.6 prolat
PLAT234_ALERT_4_C Large Hirshfeld Difference C18 --C19 . 0.16 Ang.
PLAT906_ALERT_3_C Large K Value in the Analysis of Variance ...... 2.128 Check
PLAT910_ALERT_3_C Missing # of FCF Reflection(s) Below Theta(Min). 8 Note
PLAT934_ALERT_3_C Number of (Iobs-Icalc)/Sigma(W) > 10 Outliers .. 1 Check

PLAT975_ALERT_2_C Check Calcd Resid. Dens. 1.02A From N3 0.64 eA-3
PLAT978_ALERT_2_C Number C-C Bonds with Positive Residual Density. 0 Info

Alert level G

PLAT004_ALERT_5_G Polymeric Structure Found with Maximum Dimension 2 Info
PLAT154_ALERT_1_G The s.u.'s on the Cell Angles are Equal ..(Note) 0.002 Degree
PLAT232_ALERT_2_G Hirshfeld Test Diff (M-X) Yb1 --I1 . 8.5 s.u.
PLAT232_ALERT_2_G Hirshfeld Test Diff (M-X) Yb1 --I2 . 12.5 s.u.
PLAT300_ALERT_4_G Atom Site Occupancy of C19A Constrained at 0.6 Check
And 7 other PLAT300 Alerts
More ...
PLAT301_ALERT_3_G Main Residue Disorder ....(Resd 1) 6% Note
PLAT410_ALERT_2_G Short Intra H...H Contact H17 ..H19A . 2.13 Ang.
2-x,-y,2-z = 2_757 Check
PLAT605_ALERT_4_G Largest Solvent Accessible VOID in the Structure 262 A**3
PLAT868_ALERT_4_G ALERTS Due to the Use of _smtbx_masks Suppressed ! Info
PLAT912_ALERT_4_G Missing # of FCF Reflections Above STh/L= 0.600 1557 Note

0 **ALERT level A** = Most likely a serious problem - resolve or explain
0 **ALERT level B** = A potentially serious problem, consider carefully
7 **ALERT level C** = Check. Ensure it is not caused by an omission or oversight
17 **ALERT level G** = General information/check it is not something unexpected

1 ALERT type 1 CIF construction/syntax error, inconsistent or missing data
6 ALERT type 2 Indicator that the structure model may be wrong or deficient
4 ALERT type 3 Indicator that the structure quality may be low
12 ALERT type 4 Improvement, methodology, query or suggestion
1 ALERT type 5 Informative message, check

It is advisable to attempt to resolve as many as possible of the alerts in all categories. Often the minor alerts point to easily fixed oversights, errors and omissions in your CIF or refinement strategy, so attention to these fine details can be worthwhile. In order to resolve some of the more serious problems it may be necessary to carry out additional measurements or structure refinements. However, the purpose of your study may justify the reported deviations and the more serious of these should normally be commented upon in the discussion or experimental section of a paper or in the "special_details" fields of the CIF. checkCIF was carefully designed to identify outliers and unusual parameters, but every test has its limitations and alerts that are not important in a particular case may appear. Conversely, the absence of alerts does not guarantee there are no aspects of the results needing attention. It is up to the individual to critically assess their own results and, if necessary, seek expert advice.

Publication of your CIF in IUCr journals

A basic structural check has been run on your CIF. These basic checks will be run on all CIFs submitted for publication in IUCr journals (*Acta Crystallographica*, *Journal of Applied Crystallography*, *Journal of Synchrotron Radiation*); however, if you intend to submit to *Acta Crystallographica Section C* or *E* or *IUCrData*, you should make sure that full publication checks are run on the final version of your CIF prior to submission.

Publication of your CIF in other journals

Please refer to the *Notes for Authors* of the relevant journal for any special instructions relating to CIF submission.

PLATON version of 22/12/2019; check.def file version of 13/12/2019

Datablock lv_ybi2_23_19 - ellipsoid plot

Download CIF editor (publCIF) from the IUCr
Download CIF editor (enCIFer) from the CCDC
Test a new CIF entry
