# Supplementary material for: Chemical engineering of quasicrystal approximants in lanthanide-based coordination solids
Source: Nat Commun. 2020 Sep 17;11:4705. doi: 10.1038/s41467-020-18328-5 (PMC7498582; doi:10.1038/s41467-020-18328-5)
Supplement: Supplementary file 5 — Supplementary Data 4 [file 41467_2020_18328_MOESM5_ESM.pdf]

checkCIF (basic structural check) running

Checking for embedded fcf data in CIF ...
Found embedded fcf data in CIF. Extracting fcf data from uploaded CIF, please wait . .

checkCIF/PLATON (basic structural check)

Structure factors have been supplied for datablock(s) lv_ybi_57_57_repeat

THIS REPORT IS FOR GUIDANCE ONLY. IF USED AS PART OF A REVIEW PROCEDURE FOR PUBLICATION, IT SHOULD NOT REPLACE THE EXPERTISE OF AN EXPERIENCED CRYSTALLOGRAPHIC REFEREE.

No syntax errors found.
Please wait while processing ....

CIF dictionary
Interpreting this report

Structure factor report

Datablock: lv_ybi_57_57_repeat

Bond precision:	C-C = 0.0108 A		Wavelength=0.71073
Cell:	a=9.1540(6)	b=12.1469(6)	c=14.4851(8)
	alpha=94.419(4)	beta=91.510(5)	gamma=93.165(4)
Temperature:	120 K		

	Calculated	Reported
Volume	1602.64(16)	1602.64(16)
Space group	P -1	P -1
Hall group	-P 1	-P 1
Moiety formula	C24 H24 I2 N4 O Yb, C4 H8 O [+ solvent]	C24 H24 I2 N4 O Yb, C4 H8 O
Sum formula	C28 H32 I2 N4 O2 Yb [+ solvent]	C28 H32 I2 N4 O2 Yb
Mr	883.42	883.41
Dx,g cm-3	1.831	1.831
Z	2	2
Mu (mm-1)	4.873	4.873
F000	840.0	840.0
F000'	837.65	
h,k,lmax	12,16,20	11,16,19
Nref	9091	7608
Tmin,Tmax	0.890,0.907	0.638,1.000
Tmin'	0.614	

Correction method= # Reported T Limits: Tmin=0.638 Tmax=1.000
AbsCorr = MULTI-SCAN

Data completeness= 0.837 Theta(max)= 29.704

R(reflections)= 0.0490(4630) wR2(reflections)= 0.0740(7608)

S = 0.946 Npar= 334

The following ALERTS were generated. Each ALERT has the format
test-name_ALERT_alert-type_alert-level.
Click on the hyperlinks for more details of the test.

Alert level C

PLAT213_ALERT_2_C Atom C14 has ADP max/min Ratio ..... 3.1 prolat
PLAT220_ALERT_2_C NonSolvent Resd 1 C Ueq(max) / Ueq(min) Range 3.4 Ratio
PLAT241_ALERT_2_C High 'MainMol' Ueq as Compared to Neighbors of C21 Check
PLAT243_ALERT_4_C High 'Solvent' Ueq as Compared to Neighbors of C27 Check
PLAT342_ALERT_3_C Low Bond Precision on C-C Bonds .... 0.01076 Ang.

PLAT906_ALERT_3_C Large K Value in the Analysis of Variance ...... 2.678 Check
PLAT910_ALERT_3_C Missing # of FCF Reflection(s) Below Theta(Min). 6 Note
PLAT973_ALERT_2_C Check Calcd Positive Resid. Density on Yb1 1.26 eA-3
PLAT975_ALERT_2_C Check Calcd Resid. Dens. 0.96A From N4 0.82 eA-3
PLAT977_ALERT_2_C Check Negative Difference Density on H14 -0.41 eA-3
And 3 other PLAT977 Alerts
More ...

Alert level G

PLAT004_ALERT_5_G Polymeric Structure Found with Maximum Dimension 1 Info
PLAT398_ALERT_2_G Deviating C-O-C Angle From 120 for O1 107.0 Degree
PLAT606_ALERT_4_G VERY LARGE Solvent Accessible VOID(S) in Structure ! Info
PLAT868_ALERT_4_G ALERTS Due to the Use of _smtbx_masks Suppressed ! Info
PLAT912_ALERT_4_G Missing # of FCF Reflections Above STh/L= 0.600 1342 Note
PLAT941_ALERT_3_G Average HKL Measurement Multiplicity .... 1.9 Low
PLAT978_ALERT_2_G Number C-C Bonds with Positive Residual Density. 0 Info

0 **ALERT level A** = Most likely a serious problem - resolve or explain
0 **ALERT level B** = A potentially serious problem, consider carefully
13 **ALERT level C** = Check. Ensure it is not caused by an omission or oversight
7 **ALERT level G** = General information/check it is not something unexpected

0 ALERT type 1 CIF construction/syntax error, inconsistent or missing data
11 ALERT type 2 Indicator that the structure model may be wrong or deficient
4 ALERT type 3 Indicator that the structure quality may be low
4 ALERT type 4 Improvement, methodology, query or suggestion
1 ALERT type 5 Informative message, check

It is advisable to attempt to resolve as many as possible of the alerts in all categories. Often the minor alerts point to easily fixed oversights, errors and omissions in your CIF or refinement strategy, so attention to these fine details can be worthwhile. In order to resolve some of the more serious problems it may be necessary to carry out additional measurements or structure refinements. However, the purpose of your study may justify the reported deviations and the more serious of these should normally be commented upon in the discussion or experimental section of a paper or in the "special_details" fields of the CIF. checkCIF was carefully designed to identify outliers and unusual parameters, but every test has its limitations and alerts that are not important in a particular case may appear. Conversely, the absence of alerts does not guarantee there are no aspects of the results needing attention. It is up to the individual to critically assess their own results and, if necessary, seek expert advice.

Publication of your CIF in IUCr journals

A basic structural check has been run on your CIF. These basic checks will be run on all CIFs submitted for publication in IUCr journals (*Acta Crystallographica*, *Journal of Applied Crystallography*, *Journal of Synchrotron Radiation*); however, if you intend to submit to *Acta Crystallographica Section C* or *E* or *IUCrData*, you should make sure that full publication checks are run on the final version of your CIF prior to submission.

Publication of your CIF in other journals

Please refer to the *Notes for Authors* of the relevant journal for any special instructions relating to CIF submission.

PLATON version of 04/06/2020; check.def file version of 02/06/2020

Datablock lv_ybi_57_57_repeat - ellipsoid plot

Download CIF editor (publCIF) from the IUCr
Download CIF editor (enCIFer) from the CCDC
Test a new CIF entry
